# Can vision-language models estimate patient age? evidence from panoramic radiographs

**DOI:** 10.1007/s00784-026-07183-1

**Published:** 2026-09-26

**Authors:** Thaísa Pinheiro Silva, Fernanda Bulhões Fagundes, Maria Fernanda Andrade-Bortoletto, Christiano Oliveira-Santos, Deborah Queiroz Freitas, Matheus L. Oliveira

**Affiliations:** 1 Department of Oral Diagnosis, Division of Oral Radiology, Piracicaba Dental School, University of Campinas (UNICAMP), Piracicaba, Sao Paulo 13414-903 Brazil; 2 Department of Diagnosis and Oral Health, University of Louisville School of Dentistry, Louisville, KY 40202 USA

**Keywords:** Vision-Language Models, Age Estimation, Panoramic Radiographs, Forensic Odontology, Artificial Intelligence, Radiology

## Abstract

**Objective:**

To explore the performance and limitations of three vision-language models (VLMs) in estimating chronological age from panoramic radiographs.

**Materials and Methods:**

A sample of 140 panoramic radiographs from individuals aged 7 to 20 years was uploaded to three VLMs: ChatGPT-4o, Microsoft Copilot Fast, and Microsoft Copilot Deep. Each VLM was independently prompted to estimate chronological age based on dental eruption patterns. The results were compared with the individuals’ chronological ages. Statistical analysis included agreement within ± 1 year percentage calculation, and one-way analysis of variance to compare estimated and chronological ages (α = 0.05).

**Results:**

All three VLMs demonstrated low agreement in age estimation, with overall performance below 35%. Slightly better agreement was observed in individuals aged 9–10 years. The models showed greater deviations from chronological age at the extremes of the age range (7–8 and 17–20 years). No correct estimations were recorded for individuals aged 7, 17, 18, 19, and 20 years for ChatGPT-4o, 15 and 20 years for Microsoft Copilot Fast, and 20 years for Microsoft Copilot Deep.

**Conclusions:**

The evaluated VLMs showed limited agreement in estimating chronological age from panoramic radiographs. Therefore, their application in clinical and forensic contexts cannot currently be recommended.

**Clinical Relevance:**

Understanding the potential applications and limitations of VLMs as an auxiliary tool in image interpretation is imperative to its use in clinical scenarios.

## Introduction

Dental analysis, particularly through panoramic radiographs, has long been an essential element in chronological age estimation due to the durability of teeth and their resistance to environmental factors. Traditional methods, such as those developed by Demirjian et al. and Kvaal et al., rely on assessing the development and morphological changes in teeth to estimate age. These methods have demonstrated varying degrees of agreement across different populations and age groups [1–4].

Advancements in artificial intelligence (AI) have opened new avenues for age estimation. Deep learning models, particularly Convolutional Neural Networks (CNNs), have demonstrated promising results in analyzing dental radiographs for age prediction, often achieving higher agreement and efficiency compared to traditional methods that rely on examiner-based interpretation, such as staging systems of dental development and mineralization (e.g., Demirjian or Nolla methods). These models can identify complex, non-linear patterns in dental development that may not be easily captured through conventional techniques such as visual staging of tooth mineralization. However, despite their potential, most CNN-based studies are trained on relatively homogenous datasets, which limit their generalizability across diverse populations. In addition, these models often lack transparency in decision-making, which poses challenges to their acceptance in forensic and legal contexts, where explainability and reproducibility are essential. Variability in imaging protocols, differences in dental development due to ethnicity or socioeconomic factors, and the black-box nature of deep learning architectures highlight the need for cautious interpretation and further validation of AI-based models in forensic dentistry [5, 6].

More recently, the rapid advancement of vision-language models (VLMs), such as ChatGPT (Open AI, 2023) and Microsoft Copilot (Microsoft Corporation, 2023), has expanded the potential applications of AI into new domains, including image interpretation combined with natural language understanding. These models demonstrate remarkable capabilities in processing and generating human-like text and, increasingly, in integrating visual inputs to provide contextualized responses [7–9].

Despite the growing enthusiasm surrounding AI-assisted image interpretation, significant limitations remain, particularly concerning the accuracy and reliability of outputs generated by VLMs when applied to medical imaging. One critical issue is the phenomenon referred to as hallucination, where VLMs produce confident but incorrect or misleading information unrelated to the actual input data [10]. It was demonstrated that ChatGPT-4 showed variable performance in describing radiolucent lesions on panoramic radiographs and struggled to establish accurate differential diagnoses, highlighting risks inherent in these models when applied to complex radiological tasks [11]. Furthermore, comparative studies have demonstrated substantial variability in performance among VLMs, with more advanced systems, such as ChatGPT-4o, outperforming earlier versions and alternative chatbots when responding to text-based multiple-choice questions related to oral radiology [12]. However, recent studies have also shown that VLMs may play a valuable role in dental radiology education, as VLM-generated personalized feedback has been associated with improved diagnostic performance among students [13]. Importantly, variability in performance across studies warrants caution in their use, not only in image-based diagnostics but also in VLM-generated responses to theoretical radiological content.

VLM assistants’ widespread marketing as comprehensively accurate and reliable tools can be misleading, emphasizing the dangers of uncritical adoption without sufficient domain expertise. These VLM-based systems are accessible and relatively user-friendly, which could encourage professionals to employ them in clinical or forensic contexts without fully understanding their limitations, potentially leading to diagnostic errors or legal misjudgments. In this context, it is important to recognize that VLMs are not inherently designed for quantitative image-based prediction tasks. Therefore, the aim of this study was to provide an exploratory evaluation of the performance and limitations of three VLMs in estimating chronological age based on dental development from panoramic radiographs.

## Materials and methods

### Ethical considerations and sample selection

After approval by the local institutional review board (Protocol #79179124.5.0000.5418), and in accordance with the Declaration of Helsinki, panoramic radiographs were retrospectively selected from the institution’s imaging database. The requirement for individual informed consent was waived, as all images were obtained from a pre-existing institutional repository with authorization for research use. Prior to analysis, all radiographs were anonymized to ensure the removal of any identifying information.

A total of 140 panoramic radiographs from individuals aged 7 to 20 years were included. This age range was selected to encompass key stages of dental development, including the transition from mixed to permanent dentition and third molar development, providing a clinically relevant window for dental age estimation due to ongoing tooth eruption and mineralization changes. The sample was evenly distributed across 14 age levels, with 10 radiographs per age level, including an equal distribution of sexes (5 males and 5 females per group). All images were assessed by two experienced oral and maxillofacial radiologists to ensure adequate image quality, correct patient positioning, and consistency between dental development, based on Demirjian’s method, and chronological age derived from the recorded date of birth. All panoramic radiographs were anonymized upon export in JPEG format to ensure the complete removal of any labels or metadata that could allow direct or indirect identification of the individuals. All images were exported without additional resizing or compression procedures beyond the original export settings.

### Age estimation based on VLMs

The ChatGPT-4o program (OpenAI), available at https://chat.openai.com, and the Microsoft Copilot program (Microsoft Corporation) at two operational modes (Fast and Deep), available at https://copilot.microsoft.com, were used to estimate the chronological age of each individual based on their panoramic radiograph. For this purpose, the radiographs were uploaded into the text input box of each interface, accompanied by the following prompt: “Based on this panoramic radiograph and observing the chronology of eruption, please tell me the estimated age of this patient with a margin of error of approximately one year.” The prompt was deliberately designed as a single, standardized instruction to simulate a realistic clinical scenario in which a practitioner seeks a direct age estimation based solely on the radiographic image. It was intentionally structured to elicit a global interpretation of the panoramic radiograph, reflecting the holistic approach typically adopted in dental age estimation, rather than a tooth-specific diagnostic task. The exact same prompt was applied consistently across all models and for all radiographs, without any modifications, ensuring methodological standardization and comparability of the outputs.

**Fig. 1:**
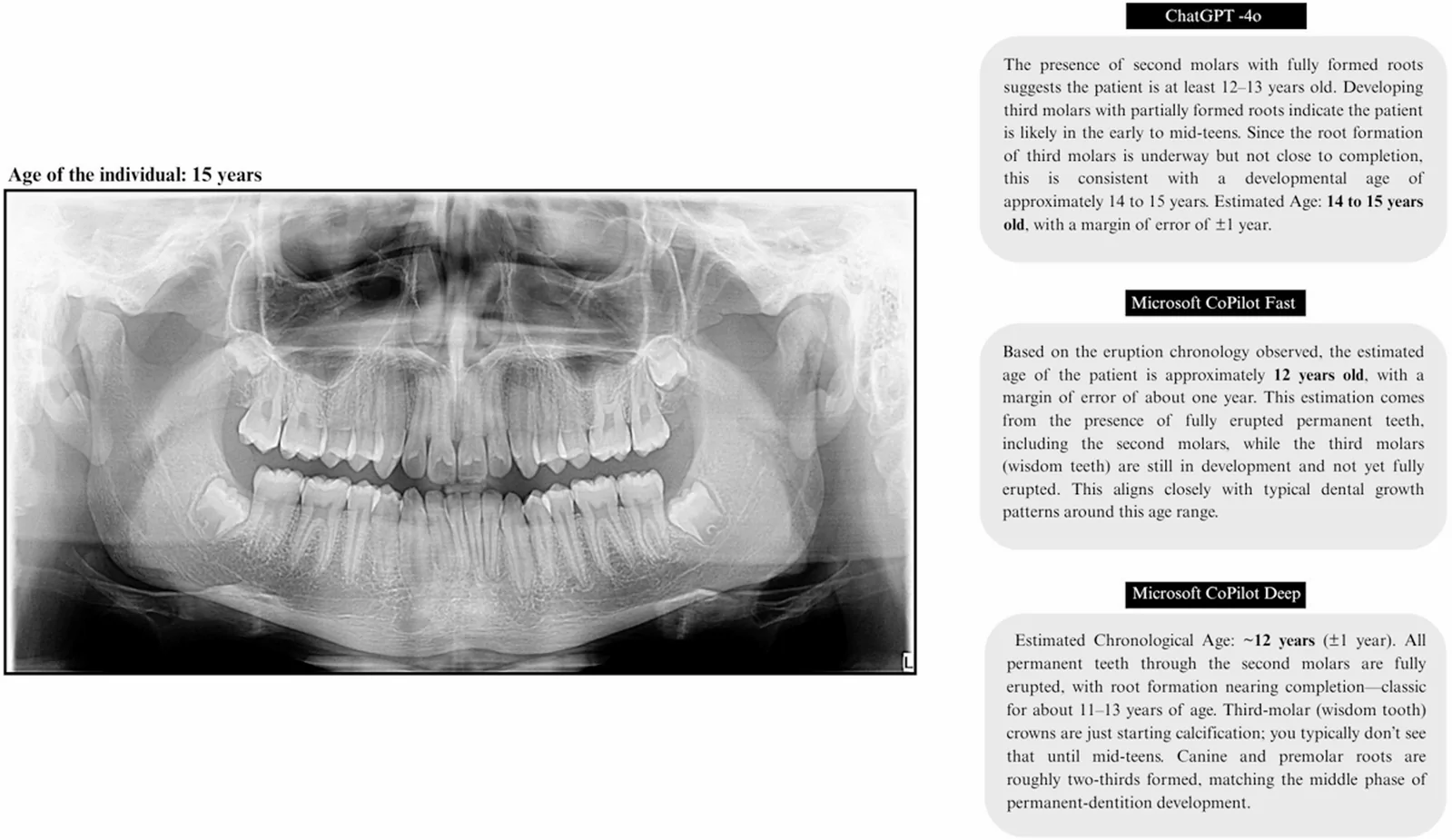
Example of full responses generated by the VLMs (ChatGPT-4, Microsoft Copilot Fast and Microsoft CoPilot Deep), based on panoramic radiography from a 15-year-old individual. The estimated age provided by each model is highlighted in bold within the output

The VLM-based systems were accessed exclusively through their public web-based platforms between June and July 2025. To ensure that each analysis was performed independently, all optional features such as web access, plugins, and memory functions were deactivated. Each panoramic radiograph was evaluated in a single, standalone interaction, with no follow-up prompts or iterative exchanges. After assessment, a new session was initiated to prevent any retention of contextual information or influence from previous cases. No additional clinical information or reference material was provided to the models. All output responses generated by the VLMs were carefully reviewed in consensus by two experienced oral and maxillofacial radiologists, who identified and extracted the keywords used by the models to estimate the individuals’ ages. Figure 1 illustrates a representative response generated by the VLMs, highlighting the estimated age.

### Data analysis

The performance of the VLMs in estimating patient age was assessed by comparing extracted keywords from the output with the chronological age. Given that age is a continuous variable, a tolerance-based approach was adopted to enhance clinical interpretability. Specifically, an estimation was considered correct when the age predicted by the VLM fell within a margin of ± 1 year from the chronological age; otherwise, it was considered incorrect. This threshold was selected because deviations within this range are generally regarded as clinically acceptable in dental age estimation. The percentage of correct age estimations was then calculated for each VLM by age level. Thus, the primary outcome measure reflects agreement within a clinically meaningful interval rather than strict exact matching. This approach allows for a practical evaluation of the model performance, aligning the analysis with real-world clinical and forensics scenarios in which small variation in estimated age may be acceptable.

Additionally, one-way Analysis of Variance (ANOVA) was conducted as a complementary inferential analysis to assess whether the mean estimated ages differed significantly from the chronological age across age groups, as well as to compare the behavior of different VLMs. This analysis was intended to identify potential systematic overestimation or underestimation patterns, rather than to evaluate agreement between predicted and true values. Dunnett’s post hoc test was applied, and a significance level of 5% (α = 0.05) was adopted. Power analysis based on the difference among the groups, their standard deviation, and the sample size yielded a statistical power of 0.9.

Confusion matrices were constructed to visualize the dispersion of age estimations performed by the VLMs by summarizing the agreement between the predicted and true age classes and displaying the distribution of correct and incorrect classifications across categories. Furthermore, to visually assess model performance across chronological age groups, a heatmap was generated based on the calculated Mean Absolute Error (MAE), which quantifies the average absolute deviation between predicted and true ages.

## Results

Table 1 presents the percentage of correct age estimation assigned to each VLM, reflecting their level of agreement with the chronological age. Overall, all evaluated VLMs demonstrated limited performance in estimating chronological age, with total agreement values below 35% for all models. Despite this generally low performance, slightly better results were observed in specific age groups, particularly between 9 and 11 years.

**Table 1:** Percentage (%) of correct age estimation by vision-language models according to chronological age

Chronological Age	Vision-Language Model		
	ChatGPT – 4o	Microsoft CoPilot Fast	Microsoft CoPilot Deep
07	00	30	20
08	40	100	40
09	70	60	60
10	60	60	70
11	70	30	30
12	40	40	50
13	50	50	30
14	30	30	40
15	10	00	10
16	10	30	10
17	00	20	30
18	00	20	30
19	00	20	10
20	00	00	00
Total	**27**	**35**	**30**

The highest agreement values were observed in isolated age groups, including 9 and 11 years for ChatGPT-4o (70%), 8 years for Microsoft Copilot Fast (100%), and 10 years for Microsoft Copilot Deep (70%). In contrast, no correct estimations were observed in several age groups, particularly at the extremes of the age range.

Mean and standard deviation of the age estimation obtained from each VLM across different chronological age levels are presented in Table 2.

**Table 2:** Mean (standard deviation) of age estimation by each vision-language model according to chronological age

Chronological Age	Vision-Languade Model			*p*-value
	ChatGPT − 4o	Microsoft CoPilot Fast	Microsoft CoPilot Deep	
7	9.85 (0.97) *	8.60 (0.81)	10.80 (2.90) *	**0.000**
8	9.15 (0.63) *	8.10 (0.81)	9.60 (1.65) *	**0.001**
9	10.20 (1.90)	8.55 (1.34)	9.70 (1.64)	0.064
10	10.50 (1.76)	9.65 (1.73)	9.10 (1.45)	0.186
11	10.45 (1.12)	9.35 (1.11) ^#^	8.60 (1.43) ^#^	**0.000**
12	13.30 (2.21)	10.85 (2.77)	10.60 (2.22)	0.392
13	13.10 (1.87)	12.45 (3.17)	12.10 (2.88)	0.749
14	12.55 (1.50)	13.90 (2.17)	14.40 (2.44)	0.130
15	11.55 (1.98) ^#^	12.55 (0.90) ^#^	12.80 (2.11) ^#^	**0.000**
16	13.45 (1.28) ^#^	14.00 (2.95)	12.80 (2.92) ^#^	**0.014**
17	12.40 (1.63) ^#^	12.90 (2.41) ^#^	13.75 (3.62) ^#^	**0.000**
18	12.60 (1.10) ^#^	13.15 (2.81) ^#^	13.90 (3.66) ^#^	**0.000**
19	13.55 (1.72) ^#^	15.40 (2.85) ^#^	13.25 (2.36) ^#^	**0.000**
20	13.25 (2.14) ^#^	15.60 (2.77) ^#^	14.70 (3.43) ^#^	**0.000**

Bold *p*-values indicate statistically significant differences within each age level. *Significantly higher than chronological age. #Significantly lower than chronological age

Significant differences among VLMs were observed in the 7-, 8-, 11-, 15-, 16-, 17-, 18-, 19-, and 20-year-old groups (*p* < 0.05). Microsoft Copilot Deep showed significant deviations from chronological age in most of the age groups (7, 8, 11, and 15 through 20 years), followed by ChatGPT-4o (7, 8, and 15 through 20 years), and Microsoft Copilot Fast (11, 15, and 17 through 20 years). No significant differences were observed either among models or in comparison to chronological age for the 9-, 10-, 12-, 13-, and 14-year-old groups (*p* > 0.05).

**Fig. 2:**
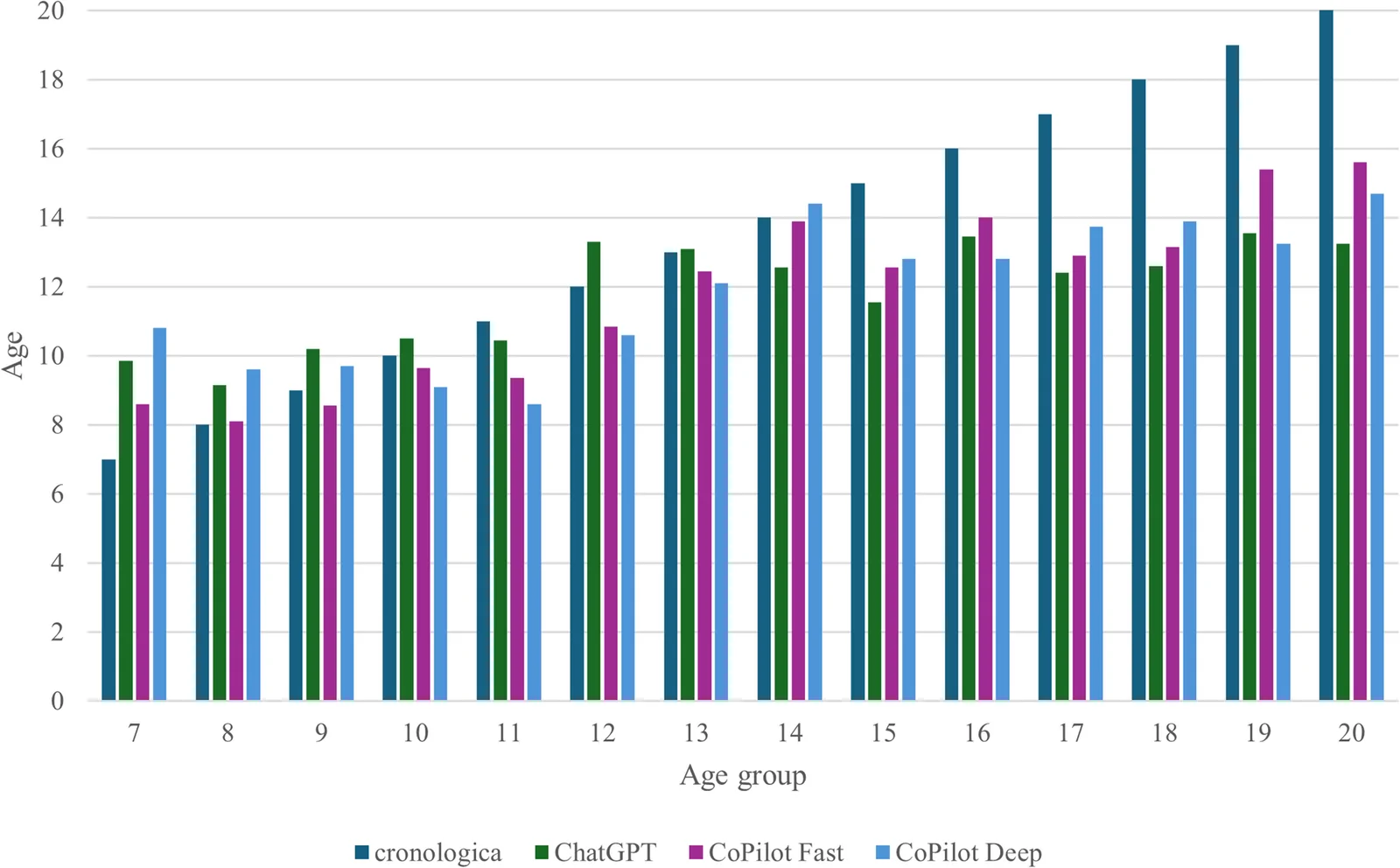
Bar graph illustrates the mean age estimations generated by each VLM in comparison to the chronological age

To provide a clearer visualization of the model’s performance across age groups, a bar graph was constructed to illustrate the mean age estimations generated by each VLM in comparison to the chronological age (Fig. 2). Overall, VLMs tended to overestimate the age of younger individuals and underestimate the age of older individuals.

**Fig. 3:**
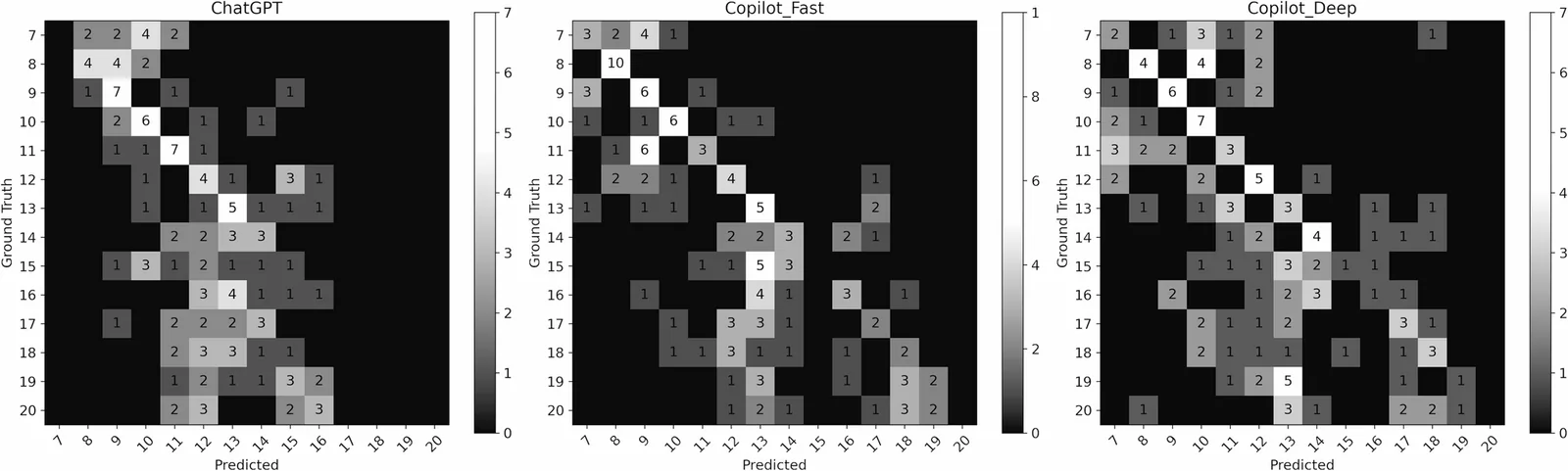
Confusion matrices for age estimation generated by ChatGPT-4o, Microsoft Copilot Fast, and Microsoft Copilot Deep

Figure 3 illustrates the confusion matrices for age estimation generated by VLMs. For ChatGPT-4o, the predicted ages for each chronological age were predominantly distributed across adjacent age categories, with a higher frequency of predictions clustered near the corresponding ground truth values. In contrast, Microsoft Copilot Fast showed a broader horizontal distribution of predicted ages for the same chronological age, spanning a wider range of age categories. Microsoft Copilot Deep exhibited the widest horizontal dispersion, with predicted ages distributed across multiple age categories for several chronological age groups.

**Fig. 4:**
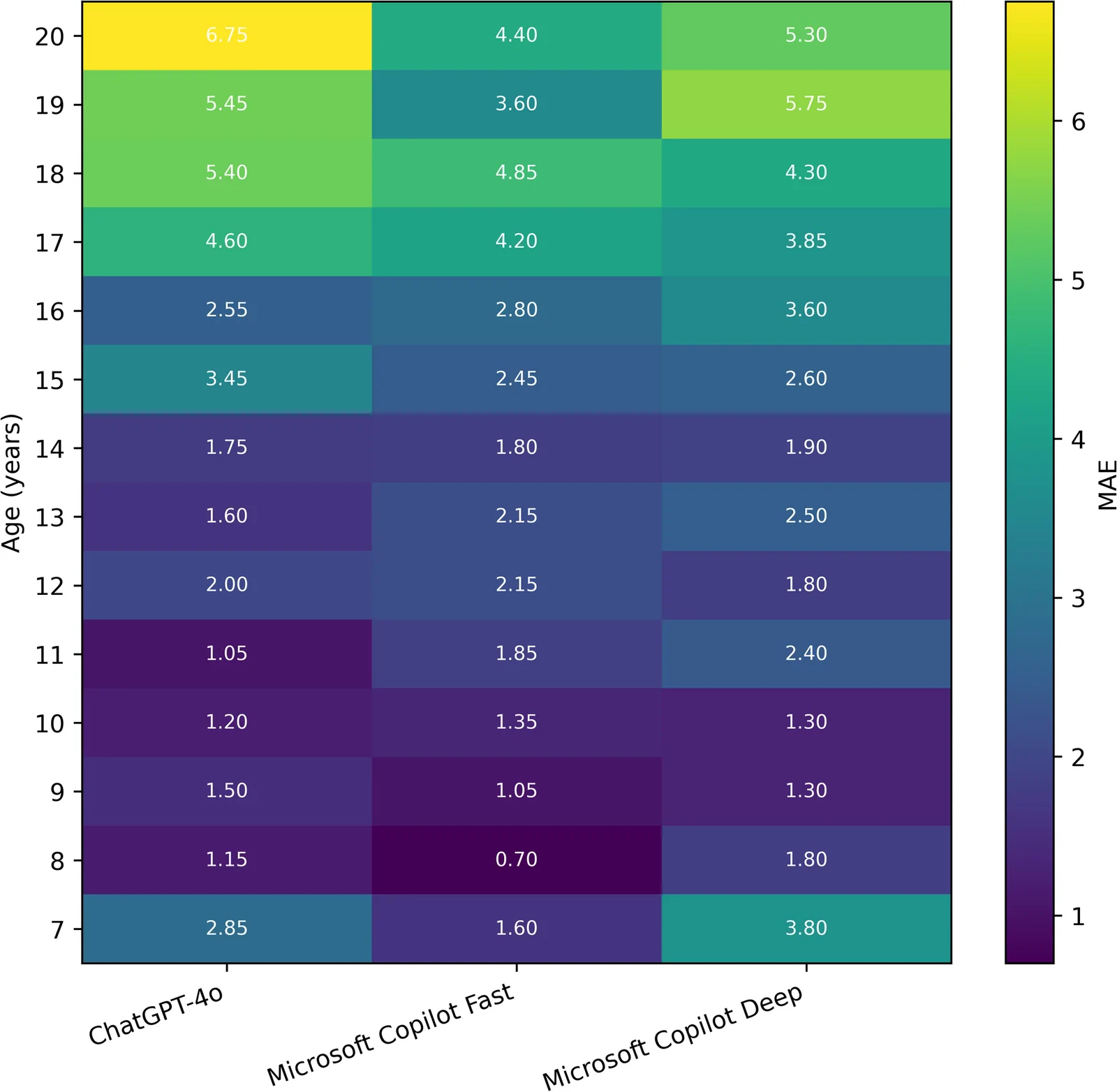
Mean absolute error (MAE) of vision-language models by age

Across all evaluated VLMs, MAE values demonstrated heterogeneity among age groups (Fig. 4). In general, lower prediction errors were observed in younger age groups, whereas higher MAE values were concentrated among older individuals. The extent of this discrepancy is represented by the heatmap’s color scale, where increasing color intensity corresponds to larger MAE values and greater deviations from chronological age.

## Discussion

This study investigated the ability of three VLMs to estimate chronological age based on panoramic radiographs. Although it has already been demonstrated that VLMs are optimized primarily for language understanding and general reasoning [14] and remain inadequate for clinical application in dentomaxillofacial radiology [15], two main reasons supported this study. First, the user-friendly interfaces and confident outputs of these models may create a false sense of reliability, encouraging users to place undue trust in their results. Second, since panoramic radiographs show extensive information on tooth development and that several age estimation methods are well documented in the literature, it was hypothesized that this task could be more readily performed by VLMs.

Overall, all three models demonstrated low mean score values of correct chronological age estimations, with a maximum inter-model difference of 3 years at the 12-year age level. Slightly higher agreement was observed in individuals aged 9–10 years, while no accurate predictions were recorded for some age groups, particularly at the lower and upper ends of the age spectrum. The limited agreement of VLMs in this context may be attributed to several factors. Unlike domain-specific AI models trained on annotated radiographic datasets, VLMs such as ChatGPT and Microsoft Copilot are primarily optimized for language understanding and general reasoning [14]. Aşar et al. [16] reported that a customized GPT-4 V model achieved 91% agreement in detecting supernumerary teeth. However, this model was specifically fine-tuned for that task. To our knowledge, this is one of the first studies to evaluate general-purpose VLMs for age estimation based solely on dental radiographic inputs, highlighting both the novelty and the experimental nature of this approach.

Interestingly, the slightly better performance in the 9 to 10-year age range may reflect a relative anatomical consistency during this stage of dental development, particularly in the mixed dentition phase. In contrast, age estimation for individuals aged 20 years was entirely inaccurate across all models. A decline in performance with increasing age was observed for ChatGPT-4o, which showed complete inaccuracy (mean score of 0) in estimating the age of individuals between 17 and 20 years. In contrast, no consistent pattern of improved or diminished performance across age levels was identified for Microsoft Copilot, in either the Fast or Deep versions. One factor that may explain this result is the absence of several developing teeth, since overall, only third molars are finishing developing. Consequently, there are a few elements to estimate age.

Regarding the statistical analysis performance of the VLMs, overall, all models exhibited substantial deviations from chronological age, particularly in older adolescents and young adults (15–20 years), where statistically significant differences were observed for most groups. Interestingly, all models achieved relatively accurate estimations in mid-childhood and early adolescence (ages 9–14), with no significant differences compared to chronological age (*p* > 0.05). These findings should not be generalized to all AI systems, particularly those specifically developed and trained for radiological image analysis, as the present study focused exclusively on general-purpose VLMs.

Similar patterns have been reported in dental age estimation research. Dursun et al. (2025) found that ChatGPT-4 achieved high agreement when guided by structured frameworks such as the London Atlas but produced systematic underestimations with the Nolla and Haavikko methods. Unlike our study, where deviations were primarily model-dependent and most pronounced at the upper end of the age spectrum, Dursun et al. (2025) emphasized the influence of the methodological approach. Taken together [17], these findings suggest that VLMs perform best in controlled methodological contexts and during developmental stages with lower biological variability, whereas their agreement declines in late adolescence or when applied without clear interpretive frameworks.

The differences observed in the horizontal distribution of the confusion matrices suggest distinct patterns of variability among the evaluated VLMs. The more constrained spread of predicted ages observed for ChatGPT-4o indicates that its estimations tend to remain closer to the chronological age, whereas the broader distributions observed for Microsoft Copilot Fast and, more markedly, for Microsoft Copilot Deep reflect greater variability in age assignment. This increased dispersion suggests a higher degree of uncertainty in the predicted age range for a given chronological age. The pattern observed suggests that performance may be influenced not only by architectural differences among VLMs but also by the biological characteristics of dental maturation. As root development nears completion and age-related morphological changes become less pronounced, the amount of radiographic information available for chronological age discrimination decreases. Consequently, even advanced multimodal models may potentially face greater challenges when estimating age in individuals with fully developed dentitions, resulting in larger deviations from the reference age.

Although the VLMs evaluated in this study are not specifically designed for radiological or forensic tasks, their integration of image-reading capabilities and their widespread commercial promotion as highly accurate, general-purpose assistants may lead clinicians to overestimate their diagnostic potential. Recent evidence indicates that while VLMs demonstrate strong performance in text-based medical tasks, their reliability declines significantly in image-dependent and quantitatively precise applications, reinforcing the need for domain-specific training and expert supervision [18]. Regarding their potential for image evaluation, similar limitations have been reported in other investigations. For example, a study assessing GPT in describing radiolucent lesions on panoramic radiographs found that its diagnostic accuracy was low, with frequent inconsistencies in differential diagnoses [11]. Camlet et al. (2025) also found that ChatGPT models 4.5 and o3 are not suitable for identifying periodontal disease using panoramic images [14]. Collectively, these findings are consistent with the present results and underscore that, without specialized adaptation, VLMs are not yet suitable for diagnostic use in dentistry or forensic sciences.

This study has limitations that should be considered when interpreting the findings. The use of a single-center dataset may limit the generalizability of the results to populations with different demographic, ethnic, or socioeconomic characteristics. Since each panoramic radiograph was evaluated a single time in the present investigation and taking into consideration the potential variability of VLM-generated outputs across repeated interactions, intra-model response consistency was not assessed, despite the use of standardized prompts. Future studies should investigate the reproducibility and stability of these systems under repeated-query conditions. Although the sample size was sufficient to achieve adequate statistical power, it remains relatively limited and restricted to individuals aged 7 to 20 years, which may further constrain broader applicability. Moreover, potential sex-related differences in dental development were not specifically analyzed. Although sexual dimorphism in dental maturation may occur, its magnitude is generally small and likely within the ± 1-year tolerance adopted in this study. Nevertheless, this factor may have a limited impact on the biological interpretability of the findings. In addition, only a limited number of general-purpose VLMs were evaluated, and the inclusion of other models, particularly those with multimodal capabilities or domain-specific adaptations, could provide additional insights. Furthermore, the models assessed in this study were not specifically trained or fine-tuned for dental radiographic interpretation, which likely contributed to their limited performance. Additionally, the use of a single, standardized prompt and a single interaction per radiograph, while ensuring methodological consistency and comparability across models, does not account for potential variability related to prompt sensitivity or stochastic output generation. Therefore, the robustness of model performance under different prompting strategies or repeated interactions was not assessed, and further investigation is warranted to better understand the stability and reliability of VLM-based outputs in clinical imaging tasks. Future research should focus on larger and more diverse datasets, alternative prompting strategies, and the development of domain-adapted models to better evaluate the potential of AI in dental age estimation.

Finally, as previously mentioned, the user-friendly interfaces and confident outputs generated by these models can create a false sense of reliability, particularly in the absence of clear disclaimers about their limitations in medical applications. This scenario underscores the importance of critical evaluation and scientific transparency regarding the actual performance of such tools. The present findings demonstrate that, at their current stage of development, these VLMs should not be relied upon for age estimation based on panoramic radiographs, and their clinical use for this purpose is not recommended without further validation and domain-specific training. Therefore, human oversight remains indispensable, particularly in tasks involving biological development and legal implications.

## Conclusion

The performance of the evaluated VLMs in estimating chronological age from panoramic radiographs was limited and age depended. While slightly better performance was observed for individuals aged 9–10 years, the models failed to provide accurate estimations for several other age groups, particularly at the extremes of the age range. Given these limitations, their application in clinical and forensic contexts cannot currently be recommended.

## Data Availability

No datasets were generated or analysed during the current study.
